# Using iDNA to determine impacts of Amazonian deforestation on *Leishmania* hosts, vectors, and their interactions

**DOI:** 10.1371/journal.pntd.0012925

**Published:** 2025-03-27

**Authors:** Aimee L. Massey, David José Ferreira da Silva, Carla Julia da Silva Pessoa Vieira, Jennifer M. Allen, Gustavo Rodrigues Canale, Christine Steiner São Bernardo, Roberta Vieira de Morais Bronzoni, Carlos A. Peres, Taal Levi

**Affiliations:** 1 Department of Fisheries, Wildlife, and Conservation Sciences, Oregon State University, Corvallis, Oregon, United States of America; 2 Instituto de Ciências da Saúde, Universidade Federal de Mato Grosso, Sinop, Mato Grosso, Brazil; 3 Instituto de Ciências Naturais, Humanas e Sociais, Universidade Federal de Mato Grosso, Sinop, Mato Grosso, Brazil; 4 Instituto Juruá, Carauari, Brazil; 5 School of Environmental Sciences, University of East Anglia, Norwich, England; Tulane University School of Public Health and Tropical Medicine, UNITED STATES OF AMERICA

## Abstract

**Background:**

There is debate concerning whether there exists a generalizable effect of land-use change on zoonotic disease risk. Strong data informing this debate are sparse because it is challenging to establish direct links between hosts, vectors, and pathogens. However, molecular methods using invertebrate-derived DNA (iDNA) can now measure species composition and interactions from vector samples at landscape scales, which has the potential to improve mechanistic understanding of the effects of land-use change on zoonotic disease risk.

**Methodology/principal findings:**

We used iDNA metabarcoding of sandflies to disentangle the relationships between *Leishmania* parasites, sandfly vectors, and vertebrate hosts. We paired these samples with iDNA metabarcoding of carrion flies to survey vertebrates independent of sandfly feeding preferences. We collected sandflies and carrion flies at forest sites across a deforestation gradient in the southern Amazon ‘Arc of Deforestation’, which exemplifies global patterns of deforestation due to agricultural expansion. We used a series of models to test whether sandflies and the vertebrate they feed upon were influenced by deforestation, which we measured using percent forest cover, percent pasture cover, and distance to the major urban center. We found that vectors were encountered less frequently in forests surrounded by pasture. We also found that the probability of a *Leishmania* host/reservoir being detected in sandfly bloodmeals was quadratically related to local forest cover, with the highest probability found at sites with intermediate levels of deforestation. Hosts were also detected most often with carrion flies at sites with intermediate forest cover, suggesting that increased host availability rather than feeding preferences was responsible for this result. Domestic dogs and the nine-banded armadillo, *Dasypus novemcinctus*, were the most prevalent hosts found in the sandfly iDNA data.

**Conclusions/significance:**

Our results did not support the generality of the ‘dilution effect’ hypothesis. However, important vectors and hosts showed consistent responses to deforestation and our findings suggest that interactions between domestic dogs and sylvatic hosts are a pathway for zoonotic disease transmission in human impacted tropical forests.

## Author summary

The effects of land-use change on disease risk is a particularly important topic in tropical forests where deforestation is concentrated and both biodiversity and disease burden are high. Vector-borne diseases require interactions among hosts, vectors, and pathogens, any of which could be either disrupted or facilitated by deforestation. We investigated how interactions between sandfly vectors and vertebrate hosts for *Leishmania* parasites respond to deforestation across a landscape defined by a matrix of forest and agriculture within the Amazonian ‘Arc of Deforestation’. The most common vector was encountered less frequently where there was more pasture. The sandflies fed on a variety of vertebrates, but domestic dogs and nine-banded armadillos were the predominant hosts. The likelihood of detecting a host in sandfly bloodmeals was highest at sites with intermediate levels of forest cover and agriculture, a pattern also observed with carrion flies, indicating that increased host availability, rather than feeding preferences, drives this result. Dogs and nine-banded armadillos were the most common vertebrate taxa in sandfly pools, suggesting the importance of both domestic and wild hosts in facilitating zoonotic disease transmission in human-impacted tropical forests.

## Introduction

Land-use change is hypothesized to be a key driver in the emergence of infectious disease [1–4]. Deforestation and the expansion of human activity into forests can alter ecological communities and interactions [5,6] and thus potentially increase the risk of infectious disease emergence from wildlife reservoirs and vectors [4,7–9]. However, there is debate on whether there is a generalizable effect of land-use patterns and consequently changes in biodiversity on increased disease risk [10–15]. The question of whether dilution, amplification, or neutralizing effects predominate for particular infectious diseases (or entire suites of pathogens) across gradients of disturbance and biodiversity is a challenging empirical problem as the mechanisms are rarely clear and often non-linear. This is particularly the case for vector-borne pathogens, which land-use change can influence by modifying the composition, density, and transmission traits of the hosts, vectors, and pathogens [16,17].

Improved mechanistic understanding of how land-use change influences host, vector, and pathogen networks is particularly critical in tropical forests where anthropogenic impacts are rapidly altering landscapes, and the burden of disease is disproportionately high. Large-scale deforestation and forest fragmentation can influence species communities by multiple mechanisms that influence pathogen dynamics and ultimately disease risk. For example, vertebrates at lower trophic levels can become hyperabundant in fragmented forest due to energy-rich subsidies from forest edges and the agricultural matrix [18–20], and due to ecological release when habitat loss and/or fragmentation leads to the decline of larger-bodied competitors and predators [21–24]. These mechanisms are supported by field surveys in the southern Amazon showing a strong negative association between the abundance of common reservoir species and forest patch size, and the extirpation of apex predators and other large-bodied taxa in the smallest forest fragments [25]. However, the relationship between forest loss and host species abundance may be nonlinear if some forest loss leads host populations to increase while continued forest loss eventually compromises the habitat integrity of vertebrate hosts, thereby leading to their decline.

While disturbance-tolerant vertebrate reservoir hosts are likely to play a key role in influencing disease prevalence and emergence as tropical forest systems are fragmented [26], this mechanism alone is not sufficient to predict how disease risk changes as tropical forests are degraded given the complex potential response of the host-vector-pathogen interaction network. For vector-borne pathogens, even if forest fragmentation increases the abundance of reservoir hosts, pathogen reproduction will be stymied if vector populations decline in edge habitats, if there is spatial mismatch between hosts and vectors, or if vectors feed disproportionately on species that are not competent reservoirs. As the context of tropical deforestation changes from agricultural expansion by many smallholders to large-scale agribusiness monoculture, the impacts of this change on vertebrate community composition may disrupt the patterns of forest loss and fragmentation on biodiversity witnessed thus far.

To tease apart the effects of large-scale land-use change on host and vector communities, and consequently disease risk, we implemented a multifaceted, landscape epidemiology approach using field-based insect trapping followed by DNA metabarcoding of sandfly vectors and their bloodmeals to disentangle the complex relationships between *Leishmania* parasites, the known sandfly vectors, and the potential wildlife hosts in response to rapid deforestation across the Amazonian ‘Arc of Deforestation’. Numerous *Leishmania* species cause cutaneous and visceral leishmaniasis [27], neglected tropical diseases that are associated with both intact tropical forest [28–30] and forest fragments [29,31]. The natural mammalian hosts are diverse, but rodents, opossums, and armadillos are thought to be strongly associated with *Leishmania* transmission [30,32–36]. Domesticated species, particularly dogs (*Canis lupus familiaris*), are also important hosts [37–39], and may play a key role as conduits of disease transmission if forest fragmentation leads to increased interactions between sylvatic and domesticated species. Given that many *Leishmania* species that cause leishmaniasis in humans are multi-host parasites [6], their prevalent transmission pathways in deforested landscapes remain an open question. Kocher et al. [17] recently found that mammal diversity, which declined with greater human footprint, was correlated with lower reservoir host abundance, lower prevalence of *Leishmania* spp. in sandflies, but high sandfly abundance. The context for this work was the intrusion of small landholders into otherwise vast, continuous tropical forest across 19 forest sites in French Guiana. Here, we use a similar landscape epidemiology approach but, in contrast to Kocher et al. [17], we sampled a gradient of forest loss and fragmentation that represents the most typical deforestation pattern witnessed across much of the Amazon and other tropical forest ecosystems worldwide, where >70% of all remaining forests is within 1 km of a forest edge [40] primarily due to agricultural expansion [41]. This intermediary stage of deforestation, where forest remnants are retained alongside large swaths of pasture or cropland, resembles the current state of tropical deforestation.

Here we use invertebrate-derived DNA (iDNA) metabarcoding of sandflies and carrion flies to assess the influence of large-scale deforestation on *Leishmania* vectors, hosts, their interactions, and pathogen prevalence. Specifically, we hypothesized that (1) sandfly abundance may either increase as a result of higher host density in degraded forests or decline due to lower quality breeding habitat by edge effects; (2) deforestation would be associated with increased bloodmeals derived from competent hosts that proliferate due to matrix subsidies and/or relaxed top-down control; and (3) interactions between domestic and sylvatic hosts will increase as forest cover decreases due to an increase in the density of domesticated species associated with the agricultural matrix. To address these hypotheses, we collected and sequenced both sandflies and carrion flies from 39 forest fragments in the southern Amazon rainforest to provide iDNA data. iDNA allows us to describe diversity using animal-feeding invertebrates such as sandflies and/or carrion flies as direct sources of species’ DNA (for more detail see Massey et al. [42]). Samples of sandflies provided us with community-level data on sandfly vectors, their potential hosts, and the prevalence of *Leishmania* species DNA. Carrion fly samples were used to provide insight on host community composition independent of the feeding preferences of sandflies since carrion flies are attracted opportunistically to scat and/or carrion. This approach allowed to determine if changes in host-vector interactions were likely due to changes in host composition (as indexed by carrion flies) or changes in sandfly feeding preferences.

## Methods

### 1. Study area

**Fig 1:**
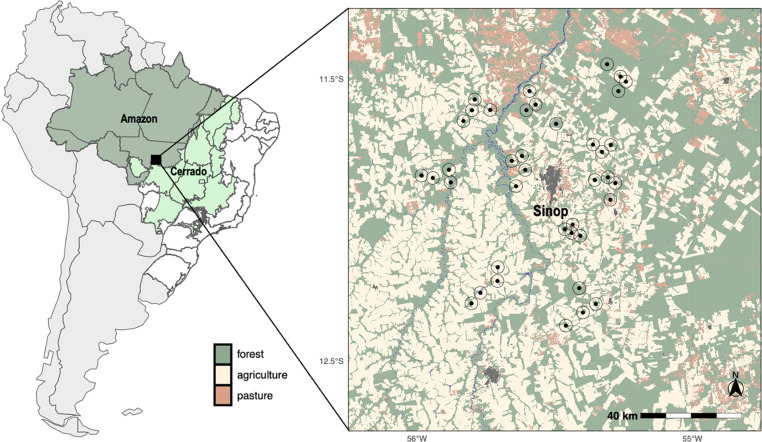
Map of the study area located near Sinop, Mato Grosso, Brazil. Sinop is an urban area (shown in dark gray on the map) with more than 160,000 people in 2015. The study region is located at the southern fringes of the Amazon forest biome, just north of the northern boundary of the Cerrado biome, and the landscape is described by a mosaic of closed-canopy forest, cerrado scrublands, croplands, and cattle pastures. Our 39 study sites and a 2500-m circular buffer are shown as solid black circles. The base layer map and the land-use, land cover classifications were sourced from MapBiomas – Collection 5 of annual series of maps of land cover and land use of Brazil, in the year 2015, accessed on 11/3/2020 through the link: https://plataforma.brasil.mapbiomas.org/.

We conducted this study near Sinop, Mato Grosso, Brazil (11.8608°S, 55.5095°W; Fig 1) within the ‘Arc of Deforestation’ at the southern edge of the Amazon forest biome. This area is largely defined by seasonally dry evergreen tropical forest (NT0140) near the transition zone between the Cerrado scrubland savannah and Amazon biomes (Fig 1). The climate is classified as neotropical with a fairly consistent mean temperature all year-round (24-25°C), but with great variation in mean precipitation between the dry (mean July rainfall ≈ 2 mm) and the wet season (mean February rainfall ≈ 309 mm). The study area was nearly completely forested until the 1970s when cattle ranching, logging, and then soybean agriculture began fragmenting the once contiguous forest and promoted rapid development of urban areas (see [43] for a history of soybean and resulting urbanization in this region of the Brazilian Amazon). This is consistent with patterns of deforestation across the Brazilian Amazon, where rapid forest clearing for pasture or cropland are the primary drivers of deforestation [44,45]. Thus, in our study landscape there is a mosaic of primarily soybean monoculture (~45%), pasture (11%), urban areas (2%), and forest remnants (~37%) that have replaced once continuous undisturbed primary forest (percentages from 10m resolution land cover data for 2016 [46]).

### 2. Fieldwork: sandfly and carrion fly collection

Fieldwork took place across 39 forest remnant sites situated within a deforestation gradient (Fig 1) and was conducted primarily during the wet-to-dry transition and dry seasons (April to August) of 2015 and 2016. We chose sites that varied in their level of deforestation and each site needed to be at least 3 km from the next closest site. At each site, we established three parallel transects 50 m apart, each with nine UV LED CDC light traps (BioQuip; Catalog Number: 2770) set 30 m apart amounting to 27 traps per site to capture sandflies. We also set 3 homemade carrion fly traps (one at each transect) to capture carrion flies (a detailed description of these carrion fly traps is available in Massey et al. [42]). We strived to set each trapping grid within the interior of the forest patch. For the smallest forest patches, the trapping grid was set in the center of the patch (equidistance from each forest edge) and at larger forest patches, the trapping grid was set at least 1 km from the closest forest edge. At each site, we captured sandflies and carrion flies over the course of three consecutive days and nights. For sandflies captured in the light traps, we collected insects after each 24-hour period and replaced each collection pot containing insects with a sterile collection pot. The collection pots from the previous 24 hours were immediately placed into a portable refrigerator in which cold temperatures immobilized the insects. We also checked each carrion fly trap for any carrion flies which were collected and immediately stored in a portable fridge. At the end of each day, insect collections were transferred to a -20°C freezer at UFMT lab facilities. At the completion of work at each individual site, sandflies were separated from other insects and stored into 2 ml Eppendorf tubes and labeled by site and date. Carrion flies were also sorted by site and date and stored in 15 ml tubes. These collections were placed in a -80°C freezer until they were shipped using dry ice to our home laboratory at Oregon State University where they were once again frozen at -80°C until molecular processing.

### 3. Landscape analysis

To formally quantify the level of deforestation at each sampled site, we first created a land cover map of our study landscape using a geotiff layer depicting land-use and land cover (LULC) of Mato Grosso, Brazil in 2015 made freely available by the MapBiomas platform [46] (Fig 1). We modified the original LULC map by reclassifying land cover classes to reduce the number of classes to forest, agriculture (including cropland and pasture), or urban. We primarily used the *raster* [47], *sp* [48,49], *sf* [50], and *terra* [51] packages in R for reading and manipulating the spatial data.

**Fig 2:**
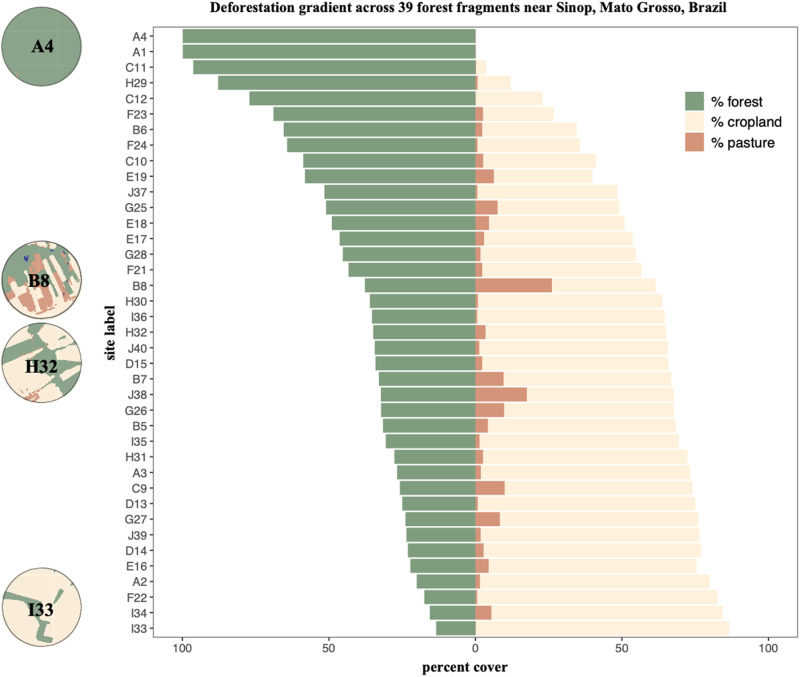
Deforestation at each site. Percentage cover of forest, cropland agriculture, and cattle pasture within a 2500-meter buffer around each trapping site. The variability of forest cover and agriculture+pasture cover showcases the deforestation gradient across the entire study landscape. Example sites from across the deforestation gradient are shown (A4, B8, H32, I33) in order of decreasing amount of forest cover.

From this LULC map, we measured the percentage cover of forest, pasture, and cropland (using the *landscapemetrics* [52] package in R) within a 2500 m circular buffer of each trapping grid (Figs 1 and 2). We selected a 2500 m buffer to capture the effects of deforestation and encroachment of non-forest cover surrounding each forest patch while also reducing the overlap between neighboring buffer windows. Percentage of forest and cropland cover within a 2500 m circular buffer around each trapping grid were highly anticorrelated (r = -0.97) so for subsequent analyses, we included only percentage forest cover in evaluating the effects of deforestation in the study region.

### 4. DNA metabarcoding of pooled sandfly samples and pooled carrion fly samples

#### Sample pooling and DNA extraction.

We sorted all captured insects based on trap date and site; sandflies were sorted into pools of 50 individuals and carrion flies sorted into pools of 5 individuals using sterilized petri dishes and forceps. Each pool was then transferred into sterile 1.7 ml tubes where sandflies were macerated in lysis buffer using bead beaters and carrion flies were manually macerated using the blunt end of a disposable tipped applicator. We then extracted DNA from each pool with the Qiagen Blood and Tissue Kit with slight modifications. Briefly, 200 ul of Buffer ATL and 20 ul of Proteinase K were added to the sample in a 1.7 ml Eppendorf tube and the sample incubated for 3-5 hours at 56°C. Post-incubation, samples were vortexed for 10 min and then purified through washing. The DNA was eluted in a final volume of 100 ul. An extraction blank was carried through the extraction process with each round of DNA extraction (~ 1 blank for every 21 samples).

#### Sandfly DNA amplification.

To describe the sandfly community, we amplified each DNA extraction in two separate reactions using the ANML primer pair from Jusino et al. [53] with the forward primer (GGTCAACAAATCATAAAGATATTGG) and the reverse primer (GGWACTAATCAATTTCCAAATCC). These primers target a 180 bp region of the COI gene and amplify a broad range of arthropods. Although the primers do not bind without mismatches to all sandflies, after species alignment for commonly found sandfly species in this region, we found that this primer pair readily amplified in sandflies and was appropriate for distinguishing sandfly species. We used primers appended with identical twin-tags unique to each sample within a library to filter out tag jumping and chimeras. PCR reactions were carried out in a volume of 15 ul using 3 ul GoTaq Green Master Mix (final concentration of 1x), 0.1 ul of GoTaq DNA Polymerase (final concentration of 0.033 u/ul), 3 ul of forward and reverse primers (final concentration of 0.2 uM), 5.48 ul of water, 0.12 ul of BSA, 0.3 ul of dNTPs, and 3 ul of DNA template. PCR cycling was as follows [53,54]: initial denaturing at 94°C for 60 sec; 5 cycles: 94°C for 60 sec, 45°C for 90 sec, 72°C at 90 sec; 35 cycles: 94°C for 60 sec, 50°C for 90 sec, 72°C for 60 sec; and a final extension of 72°C for 7 min. A no-template control was processed for each round of PCR.

#### Vertebrate DNA amplification.

To describe the vertebrate community originating from (1) any sandfly bloodmeals in the pooled sandfly samples. and (2) any blood, fecal, or carrion meals in the carrion fly samples, we amplified each pooled sample in two separate reactions using a modification of the primer pair 12SV5F/12SV5R [55]. We used the reverse primer (TTAGATACCCCACTATGC) as Riaz et al. (2011) [55] and a modified version of the forward primer to allow for broader binding of vertebrate targets (YAGAACAGGCTCCTCTAG). These primers target approximately 100 base pairs in the 12S rRNA gene region of the vertebrate mitochondrial genome. As above, we used primers appended with identical twin-tags. Briefly, we carried out PCR reactions in a volume of 20 ul using 10 ul AmpliTaq Gold 360 Master Mix (final concentration of 1x), 5 ul of forward and reverse primers (final concentration of 0.25 uM), 3 ul of water, and 2 ul of DNA template. PCR cycling was as follows: initial denaturing at 95°C for 10 min; 40 cycles: 95°C for 30 sec, 58°C for 30 sec, 72°C for 30 sec; and a final extension at 72°C for 7 min. A no-template control was processed for each round of PCR.

#### Library preparation.

After the initial PCR, we cleaned all amplicons using PCRClean DX solid-phase reversible immobilization magnetic beads (Aline Biosciences, Woburn, MA). Each PCR reaction was quantified using Accublue High Sensitivity dsDNA Quantitation kit (Biotium, Fremont, CA) and normalized to 6 ng/ul. For COI preparation, each group of 192 PCR products was pooled into a single library for a total of eight libraries (1,536 PCR products). For 12S preparation, each group of 384 PCR products was then pooled into a single library for a total of four libraries (1,536 PCR products). Individual libraries were labelled with an additional 6 base pair index using the NEBnext Ultra II DNA Library Prep kit (New England Biolabs, Ipswich, MA). Pooled samples were analyzed on a Bioanalyzer to confirm fragment size. The libraries were then sequenced using the Illumina HiSeq 3000 2 x 150 bp PE at the Center for Quantitative Life Sciences at Oregon State University.

#### Sequence analysis.

Raw sequence reads were analyzed using a bioinformatics pipeline designed to trim and sort the sequence reads according to sample and replicate identification. An outline of the bioinformatic process is as follows: (1) raw reads were paired using PEAR software [56]; (2) followed by demultiplexing using 8 basepair index sequences unique to each sample (mismatches discarded) with a novel grep regular expression (see [57]); (3) lastly, sequences were clustered to 100% similarity and then the OTUs (defined as a unique sequence) from each sample were taxonomically assigned to the best match using BLAST (www.ncbi.nlm.nih.gov/blast) against all 12S vertebrate sequences available in NCBI GenBank and using BLAST against all COI arthropod sequences available in MIDORI [58,59].

A series of filtering and quality control measures were carried out on taxonomically assigned sequences for both the sandfly taxa and vertebrate taxa datasets (workflow shown in S1 Fig). To identify the sandfly species present in each pooled sandfly sample, we removed non-amplifying samples with a 500 read threshold for each sample replicate. We then removed non-sandfly sequences based on family and genus taxonomic designations so that only sandfly species were retained. OTUs with a percentage identity score less than 96% and query sequences that totaled less than 1% of the total number of sequences in that sample were removed. Finally, species that were not present in both sample replicates were removed. With this curated dataset, we manually blasted each individual OTU to examine if there were other local taxa with equal or nearly equal query percentage matches. If so, we reassigned these species to the genus level, which was the case for all *Nyssomyia* species.

For 12S vertebrate data originating from sandfly bloodmeals or carrion fly meals, we removed OTUs with a percentage identity score less than 96% and query sequences that totaled less than 1% of the total number of sequences in that sample. We then filtered out OTUs that were identified as human DNA or likely contaminants (present in blanks or negative controls, species not regional to Brazil, and common lab contaminants). For domestic dog, which is an important *Leishmania* host species in our study region but can also be a lab contaminant, we examined each blank and negative control for the presence of dog DNA; if found, we removed all reads corresponding to dog from that set of samples. Finally, we compared taxonomic assignments with the known regional fauna to reassign non-regional species with closely related, regional matches that were equal percent identity matches. If no suitable species-level matches were discovered, these taxa were then assigned at the genus or family level or removed from the dataset.

### 5. Leishmania screening

We tested each extraction for the presence of *Leishmania* species DNA with real-time quantitative PCR (qPCR) using primer pairs kDNA1 and *L. braziliensis* kDNA3 as described by Weirather et al. [60]. We used both primers to increase the likelihood of amplifying DNA from both subgenera of the genus *Leishmania*. The kDNA1 primers were used to amplify DNA from *L. (Leishmania)*, while the *L. braziliensis* kDNA3 primers were used to amplify DNA from *L. (Viannia)* [60]. PCR reactions were carried out in a volume of 10 ul using 5 ul PowerTrack SYBR Green Master Mix (final concentration of 1x), 0.05 ul of the forward primer (final concentration of 500 nM), 0.05 ul of the reverse primer (final concentration of 500 nM), 2.9 ul of water, and 2 ul of the DNA template. The PCR cycling was as follows [60]: 95°C for 10 min; 40 cycles of 95°C for 15 sec; and 60°C for 1 min. We carried out a total of 2,260 reactions (1,130 reactions using the kDNA1 primers and 1,130 reactions using the *L. braziliensis* kDNA3 primers) to screen for *Leishmania* presence.

### 6. Data analysis

We calculated a relative abundance index (RAI) for each species in the sandfly community and the vertebrates they feed upon using frequency of occurrence at each site and across the whole landscape. The RAI of a species is equal to the sum of its occurrences divided by the total number of pooled samples. We also carried out an extensive literature search to classify each sandfly species with their currently known status as a vector, possible vector, or non-vector (see Table 1 for the classifications and references). Similarly, we carried out a literature search to designate each vertebrate species as a host, a probable host, an unlikely host, or a non-host (see Table 2). If there was no available study or inconclusive data to indicate the status of a given species, we classified that species as currently ‘unknown’.

**Table 1:** Sandfly species identified through DNA metabarcoding of pooled sandfly samples from partly deforested agricultural landscapes near Sinop, Mato Grosso, Brazil and the currently accepted vector status of each sandfly taxon.

Species	Vector Status	References
*Evandromyia walkeri*	possible*	[63–65]
*Lutzomyia longipalpis*	vector**	[66]
*Migonemyia migonei*	vector	[67–70]
*Nyssomyia* spp.	vector	[69,70]
*Psathyromyia aragaoi*	possible*	[64,71]
*Psathyromyia shannoni*	vector	[72–74]
*Psychodopygus davisi*	vector	[75–77]
*Pressatia choti*	non-vector	[78]
*Trichophoromyia auraensis*	vector	[79,80]
*Viannamyia furcata*	vector	[66,81]

* possible designation signifies that *Leishmania* spp. DNA has been detected but the sandfly species is not currently considered a vector.

** primary vector for visceral leishmaniasis and of *Leishmania infantum* in Brazil.

**Table 2:** Vertebrate taxa identified through DNA metabarcoding of pooled sandfly samples collected from forested sites across a deforestation gradient near Sinop, Mato Grosso, Brazil. Taxa are organized by their currently known host/reservoir status.

Order	Taxa	Common Name	*Leishmania* Host or Reservoir?	References
**Mammalia**				
Artiodactyla	*Bos taurus*	cattle	unlikely	[82]
	*Mazama americana*	red brocket deer	unlikely	[83]
	*Pecari tajacu*	collared peccary	unlikely	[83,84]
	*Tayassu pecari*	white-lipped peccary	unlikely	[84]
Carnivora	*Canis lupus familiaris*	domestic dog	yes	[85–87]
	*Cerdocyon thous*	crab-eating fox	yes	[6,85,88]
	*Felis catus*	domestic cat	yes	[89,90]
	*Nasua nasua*	coati	yes	[6,91,92]
	*Puma concolor*	puma	yes	[6,93]
Cingulata	*Dasypus novemcinctus*	nine-banded armadillo	yes	[6,33,94]
	Dasypus kappleri *	greater long-nosed armadillo	probable	[95,96]
	*Dasyprocta *spp.	agouti	yes	[6,91]
Chiroptera	*Micronycteris minuta*	white-bellied big-eared bat	unknown/ probable	
	*Phyllostomus hastatus*	greater spear-nosed bat	yes	[97,98]
Didelphimorphia	*Caluromys lanatus*	brown-eared woolly opossum	unknown/ probable	[99,100]
	*Didelphis *spp.	American opossum	yes	[6,34,100–103]
	*Metachirus nudicaudatus*	brown four-eyed opossum	yes	[6,94,100]
	*Micoureus demerarae*	woolly mouse opossum	probable	[100,104]
	*Philander opossum*	gray four-eyed opossum	yes	[6]
Lagomorpha	Leporidae (family)	rabbit or hare	probable	[105–107]
Perissodactyla	*Tapirus terrestris*	tapir	unknown/ no	
Pilosa	*Choloepus didactylus*	Linneaus two-toed sloth	yes	[94,108,109]
	*Tamandua tetradactyla*	lesser anteater	yes	[94,110–112]
Primates	*Ateles marginatus*	white-cheeked spider monkey	probable	[6,113,114]
	*Sapajus *spp*.*	capuchin	yes	[91,109,115,116]
	*Chiropotes albinasus*	white-nosed saki	probable	[6,109,117]
	*Plecturocebus *spp.	coppery titi monkey	probable	[114,118]
Rodentia	*Coendou prehensilis*	Brazilian porcupine	yes	[6,96,119,120]
	*Cuniculus paca*	lowland paca	yes	[102,121,122]
	*Hydrochoerus hydrochaeris*	capybara	probable	[112,123,124]
	*Mus musculus*	house mouse	yes	[125–127]
	*Oecomys* spp.	arboreal rat	probable	[128,129]
	*Proechimys* spp.	spiny rat	yes	[6,35,101,130]
	*Rattus rattus*	rat	yes	[6,36,39,102]
Aves**				
Anseriformes	*Cairina moschata*	muscovy duck	unknown/ no **the role of birds in the transmission cycles of Leishmania species is largely unknown and at this time birds are not considered reservoirs or hosts	[131,132]
Cathartiformes	*Cathartes aura*	turkey vulture		
	*Coragyps atratus*	black vulture		
Cuculiformes	*Piaya cayana*	squirrel cuckoo		
Galliformes	*Gallus gallus*	chicken		
Gruiformes	*Psophia viridis*	green-winged trumpeter		
Passeriformes	Thamnophilidae (family)	antbird		
Piciformes	*Ramphastos tucanus*	white-throated toucan		
Rheiformes	*Rhea americana*	greater rhea		
Tinamiformes	*Crypturellus undulatus*	undulated tinamou		
Trogoniformes	*Trogon viridis*	green-backed trogon		
**Amphibia**				
Anura	*Leptodactylus pentadactylus*	smoky jungle frog	unknown/ no	

* Given ongoing taxonomic revision, we refer to members of the *Dasypus kappleri*-group as *Dasypus kappleri* despite the splitting of the greater long-nose armadillos into three species (*Dasypus kappleri*, *D. beniensis*, and *D. pastasea*) because of the uncertainty with their respective range boundaries [133].

Using the *lme4* [61] package in R, we ran a series of models to test whether sandflies and the vertebrate communities they feed upon were influenced by deforestation and land use change as indexed by forest cover, pasture cover, and distance to the major urban center. We used a quasi-Poisson generalized linear model to test whether the number of sandflies collected at each site was influenced by linear and quadratic terms for percentage forest, percentage pasture, and distance to the urban center while controlling for wet to dry season phenology (Julian day). We then used binomial generalized linear mixed models with a site-level random effect to assess whether the prevalence of vectors, and then the prevalence of each of the three most common sandfly species (*Psathyromyia aragaoi* (a possible vector), *Nyssomyia* spp. (a vector), and *Psychodopygus davisi* (a vector)), was influenced by the same environmental variables. We also used binomial generalized linear mixed models with a random effect for site to assess whether the same environmental variables affected the probability that any sandfly pool contained hosts, which we subdivided into separate models for any known host, domesticated hosts, sylvatic hosts, and all hosts including probable ones. We used additional models to predict the probability of detecting non-hosts, and the five most prevalent vertebrates from sandfly bloodmeals. Finally, we counted and visually mapped the sandfly and vertebrate species present in the subset of pools that were positive for *Leishmania*.

Because sandfly bloodmeals indicate sandfly-vertebrate interactions, which are influenced by targeted feeding behavior of vectors, we additionally used carrion fly DNA metabarcoding to determine whether changes to host abundance, rather than feeding preference, were likely responsible for any observed relationships between forest cover and the probability of detecting hosts. Similar to our analysis workflow for the sandfly iDNA data, we first measured the diversity and relative abundance of all vertebrate species found with carrion fly metabarcoding data using the RAI of each species across the landscape. Again using the *lme4* [61] package in R, we then used binomial generalized linear mixed models with a random effect for site to assess the effect of linear and quadratic terms for percentage forest on the probability that a carrion fly sample contained any vertebrate species, any known host species (limited to those that overlapped with hosts found in the sandfly pools), and domestic dog, which was the most prevalent host found in the carrion fly data.

In all our models, we centered and scaled predictors to have a mean of 0 and standard deviation of 1 to compare effect sizes. We then used the R package *sjPlot* [62] to create plots from the regression model results to visualize the relationship between the beta coefficient and the significant environmental predictors on the natural scale.

## Results

### 1. Deforestation gradient

All 39 study sites sampled here were located within a forest remnant embedded within varying levels of landscape-scale forest loss and fragmentation (percent cover ranged from 13% - 100%) (Fig 2) considering the 2500-m buffer around each site. The decline of forest cover was associated primarily with increased cropland agriculture land cover (R = 0.97). Secondarily, cattle pasture was present in varying amounts across all sites (percent cover ranged from 1% - 26%) (Fig 2).

### 2. Sandfly diversity and identified vector species

We captured 56,775 sandflies and sorted them into pools of 50 flies per pool resulting in a total of 1,137 sandfly pools. The number of sandflies captured at each site ranged from 131 individuals at site E19 to 8,469 individuals at site B5 (site level data summary available in: S1 Table).

**Fig 3:**
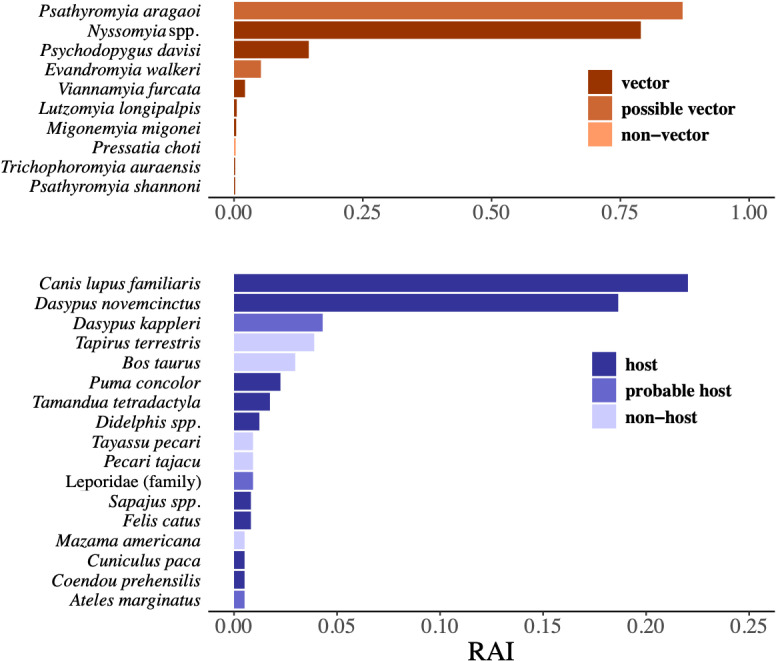
DNA metabarcoding results. Sandfly (top) and vertebrate (bottom) species diversity as revealed by metabarcoding of sandfly DNA extractions. The relative abundance index (RAI) was calculated as the total number of occurrences for species *i* divided by the total number of pooled samples from across the entire study landscape.

Metabarcoding results filtered for sandfly species (Psychodidae: Phlebotominae) revealed 34,598,149 total paired sequence reads (we retained 64% of the total number of sequences after filtering out non-sandfly taxa) from 937 pooled sandfly samples (the number of molecularly processed samples is less than the total number of samples due to the exclusion of some pooled samples from sites with disproportionately high number of sandflies; see S1 Table for details). After quality control measures designed to remove non-amplified samples and clean the raw sequencing data, the final dataset used for analysis had 22,911,864 total paired sequence reads from 864 pooled samples from across all 39 sites. These data identified 10 sandfly taxa (Table 1). We conservatively grouped all identified *Nyssomyia* species at the genus level due to multiple *Nyssomyia* species matches per OTU. All other sandfly taxa were kept at the species level. After reviewing the literature on sandfly species found in Brazil and their medical importance, we found that of the 10 sandfly taxa, seven are currently considered vectors of zoonotic disease (Table 1 and Fig 3). The most abundant sandfly species was *Pa. aragaoi* (RAI = 0.87) which is classified as a possible vector. Of all known vectors of *Leishmania* spp. from our findings, *Nyssomyia* spp. (RAI = 0.79) were the most prevalent followed by *Ps. davisi* (RAI = 0.15), both of which are vectors of cutaneous leishmaniasis. Two vectors of visceral leishmaniasis, *Lutzomyia longipalpis* and *Migonemyia migonei*, were also present but at low relative abundance (RAI < 0.01) (Table 1 and Fig 3).

### 3. Vertebrate diversity and identified host species

Metabarcoding results for vertebrate species revealed 25,093,673 total paired sequence reads from 976 pooled sandfly samples and 16,130,638 reads from 370 pooled carrion fly samples. After quality control measures designed to clean the raw sequencing data, the final dataset from sandfly samples used for analysis had 9,921,079 total paired sequence reads (human reads accounted for more than 11 million of the removed reads) from 501 pooled samples which represented 38 of the 39 sites. From carrion fly samples, the final dataset had 9,669,622 total paired sequence reads from 274 pooled samples across 38 of the 39 sites.

From the sandfly samples, the sequencing data revealed 46 vertebrate taxa, 44 of which were identified to the genus or species level (Table 2 and Fig 3). Of the 46 identified vertebrate taxa, 39 are considered sylvatic species and seven are domesticated species. We categorized the vertebrate taxa based on their known host and/or reservoir status for *Leishmania* parasites after an extensive review of the literature (Table 2). The most abundant vertebrate species in sandfly pools were: dogs (*Canis lupus familiaris* (RAI = 0.22)), armadillos (*Dasypus novemcinctus* (RAI = 0.19) and *Dasypus kappleri* (RAI = 0.04)), tapir (*Tapirus terrestris* (RAI = 0.04)), cattle (*Bos taurus* (RAI = 0.03)), puma (*Puma concolor* (RAI = 0.02)), and lesser anteater (*Tamandua tetradactyla* (RAI = 0.02)). Host and/or reservoir species dominated the species diversity with 29 species categorized as either host or probable host and only 4 species were categorized as non-host or unlikely host (Table 2). Too little is currently known about the host status of the remaining 15 vertebrate taxa, most of which were birds (n=11); tapir, brown-eared woolly opossum, white-bellied big-eared bat, and smoky jungle frog are the non-bird species designated as ‘unknown’ (Table 2).

**Fig 4:**
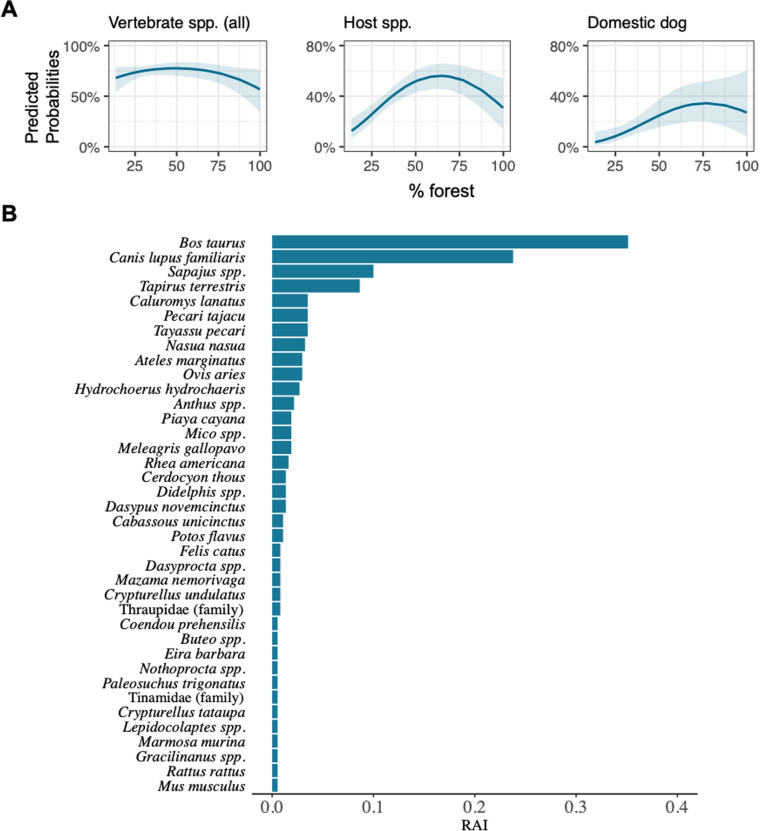
Model results for vertebrate species from carrion fly iDNA dataset. **(A)** The predicted probabilities for the significant models for all species (left panel), host species (middle panel; defined as host species in common with host species from the sandfly data), and domestic dogs (right panel) revealed by carrion fly DNA metabarcoding. **(B)** Vertebrate species diversity as revealed by metabarcoding of carrion fly DNA extractions. The relative abundance index (RAI) was calculated as the total number of occurrences for species *i* divided by the total number of pooled samples from across the entire study landscape.

After sequence cleaning, carrion fly samples revealed 56 vertebrate taxa. Of these 56 taxa, there were 29 taxa in common (and 15 of these taxa are classified as hosts) with vertebrate results from sandfly samples (Table 3). Similarly to sandfly data, carrion fly data showed a high relative abundance of cattle (RAI = 0.35), dog (RAI = 0.24), and tapir (RAI = 0.09) (Fig 4). Other abundant vertebrates include capuchin (*Cebus* spp. (RAI = 0.10), white-lipped peccary (*Tayassu pecari* (RAI = 0.04)), collared peccary (*Pecari tajacu* (RAI = 0.04)), and brown-eared woolly opossum (*Caluromys lanatus* (RAI = 0.04)) (Fig 4).

**Fig 5:**
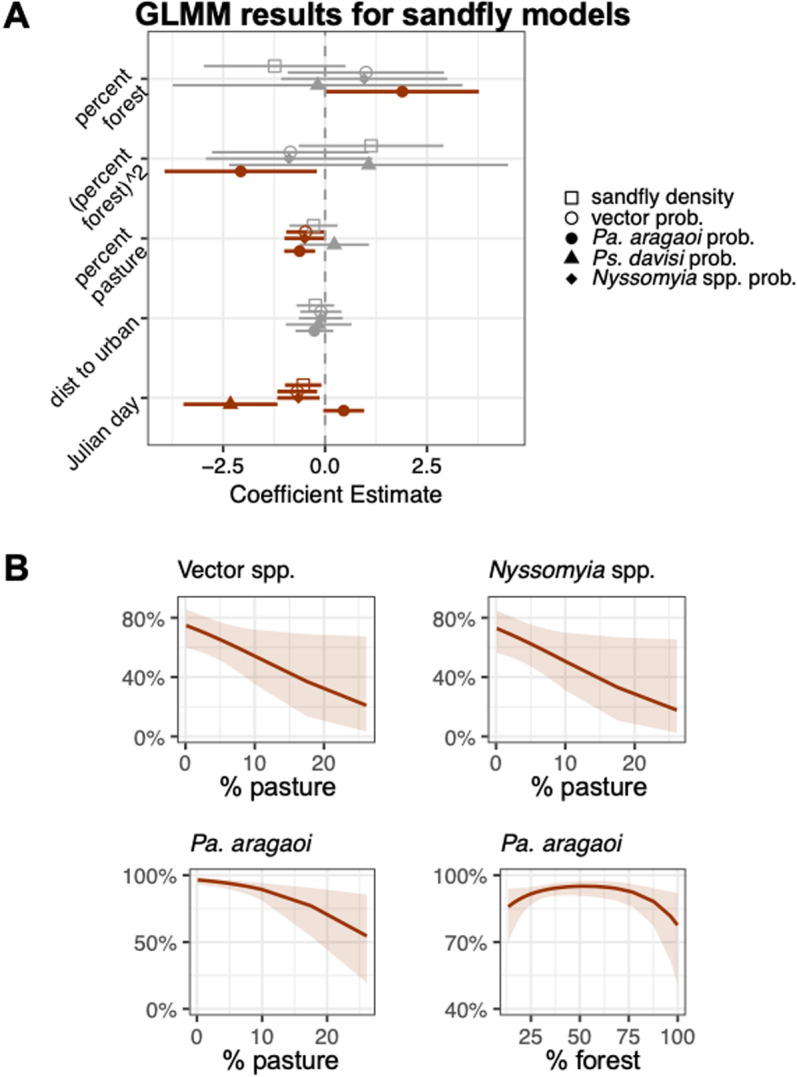
Model results for sandfly species. **(A)** Regression coefficients and their 95% confidence intervals for the effects of percentage forest (quadratic term), percentage pasture, distance to urban center, and Julian day on sandfly density (quasipoisson model) and the probability of encountering (binomial models each with a random effect for site) a vector species, *Psathyromyia aragaoi*, *Psychodopygus davisi*, and *Nyssomyia* spp. in a pooled sample of sandflies. Significant results are bolded and colored. **(B)** The significant model predicted probabilities for finding a vector, *Pa. aragaoi*, and *Nyssomyia* spp. (the significant results) in a sandfly pool across increasing pasture cover.

**Table 3:** List of vertebrate diversity revealed from carrion fly DNA metabarcoding.

Host taxa from carrion fly data in common with sandfly data		
*Canis lupus familiaris* (domestic dog)	*Dasyprocta* spp. (agouti)	*Mus musculus* (house mouse)
*Cebus* spp. (capuchin)	*Dasypus novemcinctus* (nine-banded armadillo)	*Nasua nasua* (South American coati)
*Cerdocyon thous* (crab-eating fox)	*Didelphis* spp. (American opossums)	*Phyllostomus hastatus* (greater spear-nosed bat)
*Coendou prehensilis* (Brazilian porcupine)	*Felis catus* (domestic cat)	*Rattus rattus* (black rat)
*Cuniculus paca* (lowland paca)	*Metachirus nudicaudatus* (brown four-eyed opossum)	*Tamandua tetradactyla* (southern tamandua)
**Other taxa from carrion fly data in common with sandfly data**		
*Ateles marginatus* (white-cheeked spider monkey)	*Crypturellus undulatus* (undulated tinamou)	*Plecturocebus* spp. (coppery titi monkey)
*Bos taurus* (cattle)	*Hydrochoerus hydrochaeris* (capybara)	*Tapirus terrestris* (tapir)
*Caluromys lanatus* (brown-eared woolly opossum)	*Mazama americana* (red brocket)	*Tayassu pecari* (white-lipped peccary)
*Cathartes aura* (turkey vulture)	*Pecari tajacu* (collared peccary)	*Trogon viridis* (green-backed trogon)
*Coragyps atratus* (black vulture)	*Piaya cayana* (squirrel cuckoo)	
**Taxa unique to carrion fly data**		
*Amazona* spp. (Amazon parrots)	*Gracilinanus* spp. (Gracile mouse opossum)	*Nyctiphrynus ocellatus* (ocellated poorwill)
*Anthus* spp. (pipits)	*Lepidocolaptes* spp. (woodcreeper)	*Ovis aries* (domestic sheep)
*Buteo* spp. (buzzards and hawks)	*Leporinus* spp. (Leporinus fish)	*Paleosuchus trigonatus* (smooth-fronted caiman)
*Cabassous unicinctus* (southern naked-tailed armadillo)	*Makalata didelphoides* (Brazilian spiny tree-rat)	*Potos flavus* (kinkajou)
*Caluromys philander* (bare-tailed woolly opossum)	*Marmosa murina* (Linnaeus’s mouse opossum)	*Pseudoryzomys simplex* (Brazilian false rice rat)
*Crotophaga ani* (smooth-billed ani)	*Mazama nemorivaga* (Amazonian brown brocket)	*Speothos venaticus* (bush dog)
*Crypturellus tataupa* (tataupa tinamou)	*Meleagris gallopavo* (turkey)	Thraupidae (family) (tanager)
*Dactylomys dactylinus* (Amazon bamboo rat)	*Mico* spp. (marmosets and tamarins)	Tinamidae (family) (tinamou)
*Eira barbara* (tayra)	*Nothoprocta* spp. (tinamou)	
**Taxa unique to sandfly data**		
*Cairina moschata* (Muscovy duck)	*Leptodactylus pentadactylus* (smoky jungle frog)	*Psophia viridis* (green-winged trumpeter)
*Chiropotes albinasus* (white-nosed saki)	*Micoureus demerarae* (woolly mouse opossum)	*Puma concolor* (puma)
*Choloepus didactylus* (Linnaeus’s two-toed sloth)	*Micronycteris minuta* (white-bellied big-eared bat)	*Ramphastos tucanus* (white-throated toucan)
*Dasypus kappleri* (greater long-nosed armadillo)	*Oecomys* spp*.* (arboreal rat)	Thamnophilidae (family) (antbird)
*Gallus gallus* (chicken)	*Philander opossum* (gray four-eyed opossum)	
Leporidae (family) (rabbits and hares)	*Proechimys* spp*.* (spiny rat)	

### 4. Species assemblages across the deforestation gradient

**Fig 6:**
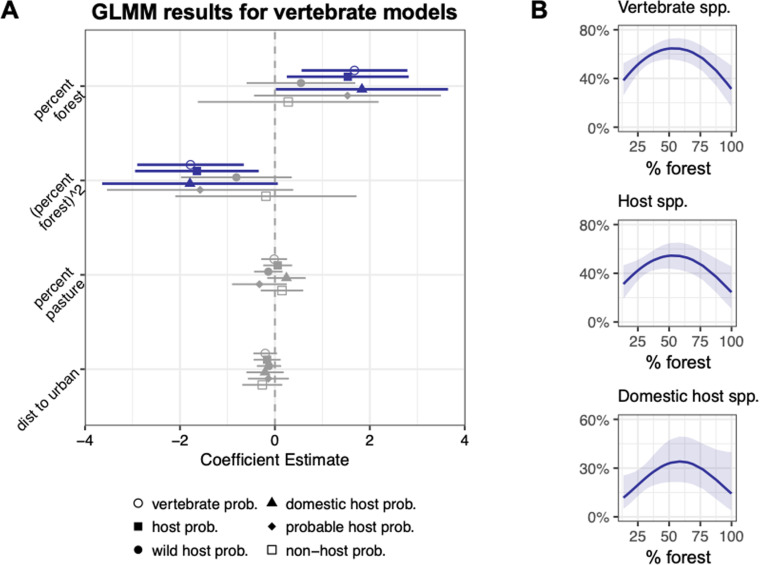
Model results for vertebrate groups from sandfly iDNA dataset. **(A)** Regression coefficients and their 95% confidence intervals for the effects of percentage forest (quadratic term), percentage pasture, and distance to urban on the probability of encountering (binomial models each with a random effect for site) a host, a sylvatic host, a domestic host, a probable host, and a non-host from a pooled sample of sandflies. Significant results are bolded and colored. **(B)** The predicted probabilities for the significant models of finding host species, domestic hosts species, and probable host species across increasing forest cover.

**Fig 7:**
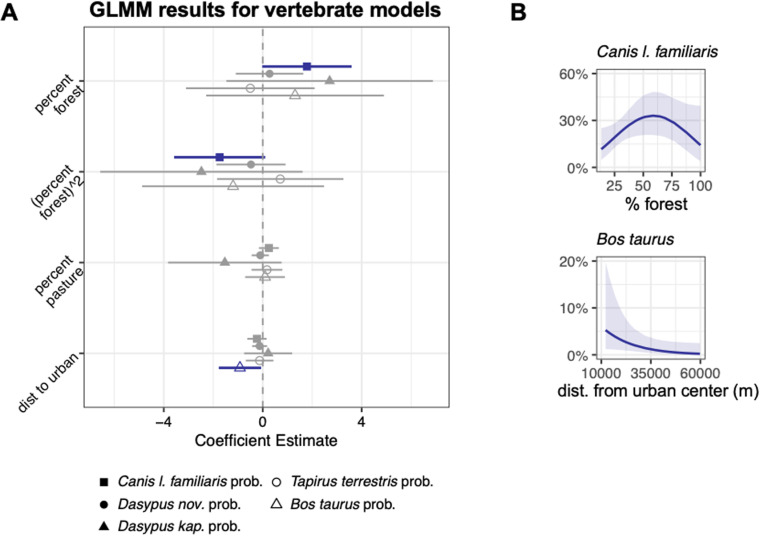
Model results for vertebrate species from sandfly iDNA dataset. **(A)** Regression coefficients and their 95% confidence intervals for the effects of percentage forest (quadratic term), percentage pasture, and distance to urban on the probability of encountering (binomial models each with a random effect for site) a target species from a pooled sample of sandflies. Significant results are bolded and colored. **(B)** The predicted probabilities for the significant models of finding *Canis lupus familiaris* (domestic dog) from a pooled sample of sandflies across increasing forest cover and *Bos taurus* (domesticated cattle) from a pooled sample of sandflies with increasing distance from the urban center of Sinop.

Regression models more clearly show the responses of species groups and individual species to deforestation (Figs 4-7 and Tables 4-6). While there was no significant relationship between any of the deforestation metrics and the density of sandflies captured at a site, we found that higher pasture land cover was a significant negative predictor of the probability of encountering a medically-important vector in general (p = 0.05), the vector genus *Nyssomyia* spp. (p = 0.05), as well as the most prevalent sandfly species *Pa. aragaoi* (p = 0.001) (Fig 5 and Table 4). *Pa. aragaoi* also showed a significant quadratic response to percentage forest with the highest probability of encountering this species occurring at intermediate amounts of forest cover (p = 0.03; Fig 5 and Table 4).

**Table 4:** Regression results with model estimates (and standard errors shown in parentheses) for sandfly models measuring individual species or species group level responses to percent forest, percent pasture, distance to the urban center, and Julian Day. Independent variables have been scaled. Significant estimates are bolded.

	percent forest	(percent forest)^2^	percent pasture	dist. to urban	Julian day
*Dependent variables*					
sandfly density	-1.235 (0.887)	1.129 (0.904)	-0.284 (0.301)	-0.237 (0.235)	-0.533 * **(0.228)**
vector probability	1.00 (0.979)	-0.852 (0.981)	-0.480 * **(0.240)**	-0.102 (0.275)	-0.682 ** **(0.250)**
*Pa. aragaoi* probability	1.893 * **(0.959)**	-2.067 * **(0.953)**	-0.624 ** **(0.194)**	-0.263 (0.238)	**0.457.** **(0.255)**
*Ps. davisi* probability	-0.182 (1.812)	1.069 (1.745)	0.225 (0.436)	-0.157 (0.411)	-2.32 *** **(0.588)**
*Nyssomyia* spp. probability	0.960 (1.040)	-0.883 (1.041)	-0.500 * **(0.254)**	-0.104 (0.273)	-0.653 * **(0.265)**

Significance is as follows:. p<0.1; * p < 0.05; ** p<0.01; *** p<0.001

The vertebrate responses to deforestation included a consistent quadratic relationship indicating that the probability of detecting a host (p = 0.01), a domestic host (p = 0.06), domestic dogs (p = 0.06), or any vertebrate (p = 0.002) in sandfly bloodmeals was highest at intermediate forest cover (Figs 6 and 7 and Table 5). In contrast, the probability of detecting cattle increased as the distance from the urban center decreased (p = 0.04; Fig 7 and Table 5).

**Table 5:** Regression results with model estimates (and standard errors shown in parentheses) for vertebrate models measuring individual species or species group level responses to percent forest, percent pasture, and distance to the urban center. Independent covariates have been scaled and centered. Significant estimates are bolded.

	percent forest	(percent forest)^2^	percent pasture	dist. to urban
*Dependent variables*				
vertebrate probability	1.676 ** **(0.569)**	-1.778 ** **(0.573)**	-0.016 (0.139)	-0.204 (0.127)
host probability	1.535 * **(0.655)**	-1.643 * **(0.665)**	0.059 (0.155)	-0.164 (0.145)
wild host probability	0.549 (0.584)	-0.811 (0.595)	-0.139 (0.153)	-0.124 (0.130)
domestic host probability	1.833 * **(0.926)**	**-1.790.** **(0.944)**	0.243 (0.207)	-0.207 (0.200)
probable host probability	1.530 (1.00)	-1.58 (1.00)	-0.326 (0.294)	-0.137 (0.220)
non-host probability	0.333 (0.975)	-0.267 (0.979)	0.146 (0.229)	-0.268 (0.215)
*Canis lupus familiaris*	**1.786.** **(0.921)**	**-1.741.** **(0.939)**	0.249 (0.205)	-0.234 (0.200)
*Dasypus novemcinctus*	0.277 (0.697)	-0.474 (0.713)	-0.100 (0.177)	-0.126 (0.154)
*Dasypus kappleri*	2.705 (2.129)	-2.478 (2.087)	-1.532 (1.171)	0.200 (0.500)
*Tapirus terrestris*	-0.502 (1.326)	0.708 (1.303)	0.170 (0.318)	-0.125 (0.284)
*Bos taurus*	1.307 (1.833)	-1.199 (1.876)	0.094 (0.411)	-0.920 * **(0.437)**

Significance is as follows:. p<0.1; * p < 0.05; ** p<0.01; *** p<0.001

Vertebrate detections in carrion fly iDNA also followed a quadratic relationship for all species combined (p = 0.09), vertebrates classified as host species (defined as host species that overlapped with host species found with sandfly samples; p < 0001), and dogs (p = 0.09) (Fig 4 and Table 6).

**Table 6:** Regression results with model estimates (and standard errors shown in parentheses) for carrion fly models measuring individual species or species group level responses to percent forest. Independent variables have been scaled.

	percent forest	(percent forest)^2^
*Dependent variables*		
all vertebrates probability	0.883 (0.594)	**-1.025.** **(0.606)**
host probability	2.553 *** **(0.630)**	-2.270 *** **(0.641)**
*Canis lupus familiaris*	2.367 * **(1.068)**	**-1.764.** **(1.043)**

Significance is as follows:. p<0.1; * p < 0.05; ** p<0.01; *** p<0.001

### 5. Leishmania incidence

**Fig 8:**
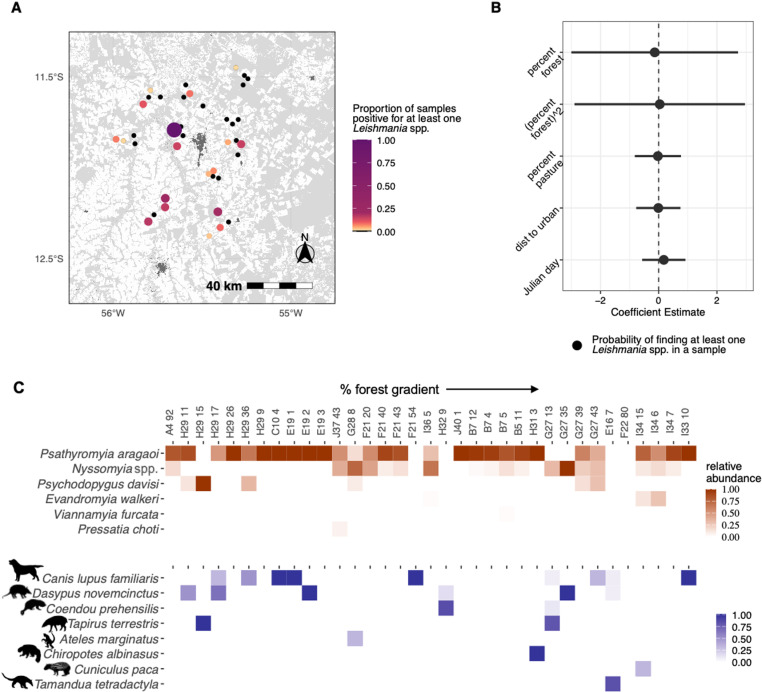
Leishmania results. **(A)** Locations of *Leishmania* positive sites across our study landscape. Size and color of each circle indicate the proportion of samples at that site that tested positive for *Leishmania* species with either the kDNA1 primer or the *L. braziliensis* kDNA3 primer. The black circles indicate sites where there were no samples that tested positive for *Leishmania*. The base layer map and the land-use, land cover classifications were sourced from MapBiomas – Collection 5 of annual series of maps of land cover and land use of Brazil, in the year 2015, accessed on 11/3/2020 through the link: https://plataforma.brasil.mapbiomas.org/. **(B)** Regression coefficients and their 95% confidence intervals showing the effect of percent forest, percent pasture, distance to the urban center, and Julian Day on the probability of a pooled sample of sandflies testing positive for the presence of *Leishmania*. **(C)** Of 1130 samples, 42 were positive for the presence of at least one *Leishmania* species, 35 of which containing either sandfly and/or vertebrate data from DNA metabarcoding. The samples that tested positive for *Leishmania* are organized on the horizontal axis by amount of forest cover at their corresponding site. The sandfly and vertebrate taxa that were found in these samples are shown with either orange (sandfly) or purple (vertebrate) color showing the relative abundance of sequence reads for each taxon in that sample.

Of the 1,130 total sandfly pools screened for the presence of *Leishmania*, 42 samples tested positive for the presence of at least one *Leishmania* species (3.7% positivity rate). Given that only female sandflies of some species transmit *Leishmania* parasites, this positivity rate was likely influenced by the inclusion of all sandfly species and of males in our sandfly pools. The proportion of infected pools at a site varied substantially (S2 Table) across all sites but there was no significant effect of any of the measured environmental metrics on the likelihood of a pool testing positive for *Leishmania* (Fig 8). Eighteen of the 39 sites had at least one infected pool, with one site (E19) having 100% of pools (n=3) infected. Aside from this outlier site, sites with infected pools had positivity rates that ranged from 0.01 – 0.20. Of the 42 positive samples, 17 samples contained vertebrate DNA. In those samples, dogs (n=9) and armadillos (n=6) were the most frequently detected taxa and four samples had co-occurrences of multiple vertebrate species (at least two vertebrate species found in the same sample) (Fig 8).

## Discussion

There has been a robust debate about whether there is a generalizable effect of land-use change and consequent declines in biodiversity on the emergence and prevalence of infectious diseases [10–15,134]. While studies have demonstrated some of the effects of land-use change on host species [135–137], less work has been done to simultaneously examine the effects of deforestation on vectors, hosts (both sylvatic and domestic), and pathogens across different types of deforestation gradients, particularly in the tropics where poorly known infectious diseases are most prevalent. This debate has been stymied by the complexities in measuring and quantifying the mechanisms leading to changing disease risk. This can be especially complex for vector-borne pathogens where land-use change can differentially influence hosts, vectors, and pathogens [16]. We addressed this debate with a landscape epidemiology approach using DNA metabarcoding to directly detect sandfly vectors, hosts from sandfly bloodmeals, and *Leishmania* DNA across a forest loss gradient within the world’s largest tropical deforestation frontier.

By using DNA metabarcoding of sandflies and their vertebrate bloodmeals, we were able to link medically important vectors and hosts to deforestation at large scales (56,775 sandflies collected from 3,159 trap nights; 27 light traps set for 3 nights at each of the 39 sites). Metabarcoding allowed us to identify thousands of sandflies at the species or genus level, which is usually only possible with painstaking morphological identification by taxonomic experts. It is important to note, however, that the reference databases we use to taxonomically assign sequencing data rely on experts to identify specimens and deposit new sequences. Consequently, references databases for regions that are both understudied and highly biodiverse are still incomplete. Additionally, while pooling sandflies for metabarcoding was necessary to reduce costs in order to facilitate a large-scale landscape-scale epidemiology approach, this came at the cost of not being able to construct precise host-vector interaction networks due to data from multiple sandfly species occurring in the same pool. However, pooling sandflies for metabarcoding allows for taxonomic identification of vectors and hosts, which allows for their prevalence to be modeled independently as a function of landscape variables.

Another limitation of using iDNA data from vectors is that the vertebrate diversity described from their bloodmeals is influenced by both the feeding preferences of said vectors and the patterns of occurrence or relative abundance of vertebrates. Our solution to this potential problem was to pair the sandfly iDNA data with carrion fly iDNA data collected from the same study sites. Carrion flies offer a less biased description of vertebrate diversity because their feeding is likely driven by the presence of scat and/or carrion [42,138,139] rather than attraction to particular species. Our previous research in this same study region showed that carrion flies better describe landscape-level biodiversity compared to the more conventional method of camera trapping [42]. Carrion fly iDNA data thus provided patterns of vertebrate prevalence that were both robust and independent of the feeding preferences of sandflies.

iDNA metabarcoding of sandflies and carrion flies revealed that this intensified agricultural landscape still supports a large diversity of terrestrial and arboreal vertebrate species, even though only a third of the original forest had been retained across the study landscape at the time of sampling. While the sandfly and carrion fly data overlapped in many of the described species, including a high relative abundance of domestic dogs detected with both methods, carrion fly iDNA contained more non-host species including a higher relative abundance of cattle and tapir, which is likely reflective of both the higher cumulative biomass of these two species and potentially by preferential feeding of sandflies on hosts for *Leishmania* spp.

In contrast, with the sandfly iDNA data we found that the responses of vertebrate taxa to deforestation were driven by the high prevalence of both domestic dogs (*Canis lupus familiaris*) and armadillos in the genus *Dasypus,* particularly the disturbance-tolerant nine-banded armadillo, *D. novemcinctus* (Fig 3). These two taxa are known hosts of various *Leishmania* species that can cause leishmaniasis in humans. Domestic dogs are a key reservoir host of *Leishmania infantum*, which can lead to visceral leishmaniasis in dogs and humans. Historically, visceral leishmaniasis stemming from *L. infantum* infection has been associated with both dense and degraded forest [140]. *L. infantum* has many other mammalian hosts [6] suggesting that *L. infantum* can make its way from the forest into developed areas through increasing proximity of sylvatic wildlife and domestic dogs. Dogs are also a known host of *Leishmania braziliensis*, which causes mucocutaneous leishmaniasis throughout much of South America and again has many other sylvatic mammalian hosts. We screened for both of these *Leishmania* species and found that dogs were the most prevalent host in the subset of samples that tested positive for the presence of *Leishmania* DNA. These findings complement recent research that has found that dogs in close proximity with sylvatic species increased overall *Leishmania* infection in dogs [141].

The nine-banded armadillo was the second most prevalent host species found in the sandfly iDNA data, including the subset of samples that tested positive for *Leishmania* DNA. Here, metabarcoding of sandfly pools at landscape scales provided direct evidence for consistent and strong selection of armadillos by sandflies. Armadillos are among the important sylvatic hosts for *Leishmania* species including *Leishmania (Viannia) naiffi*. *L. (V.) naiffi* is known to circulate in environments with varying levels of anthropogenic impact and can cause cutaneous and mucocutaneous leishmaniasis in humans [33,142]. However, the role of armadillos as important hosts for other disease-causing *Leishmania* species is still largely unknown. Much of our foundational understanding of *Leishmania* in armadillos was carried out by Lainson and others in the 1970s and 1980s [32,33], and since this time there has been a lack of further research. Our research [42] along with that of Kocher et al. [143] in French Guiana have both independently tied armadillos to sandfly bloodmeals.

Aside from domestic dogs and the nine-banded armadillo, the iDNA data from our study sites revealed an impressive diversity of known hosts as well as other probable hosts. We examined how deforestation impacts the interactions between hosts and vectors that are critical to pathogen transmission. The deforestation frontier across the southern Amazon exemplifies the current state of tropical deforestation with both extensive, rectangular-shaped clear cutting for cropland monoculture alongside irregularly shaped patches of cattle pasture. This has resulted in a landscape defined by a mosaic of juxtaposed forest and agriculture (see Figs 1 and 2). Using the metabarcoding data from sandfly bloodmeals, we found a strong and consistent quadratic response of host species to the amount of forest cover such that intermediately deforested sites had the highest probability of encountering a host species (Fig 6). When we ran this same model with the same group of hosts but using vertebrate data gleaned from carrion fly iDNA, we found the same significant quadratic relationship between hosts and percent forest (Fig 4). This suggests that heavily fragmented forests lead to increased abundance of *Leishmania* hosts relative to both intact forest and near complete deforestation, which therefore increases the probability that sandflies will feed on a competent host in fragmented forest. Our results suggest that hosts for *Leishmania* parasites (and likely other zoonotic parasites) are disturbance tolerant and have a greater relative abundance in fragmented forests.

Disease risk is a product of both host and vector population ecology, so we also examined sandfly vector diversity and responses to deforestation. We found no significant response of sandfly density or probability of encountering a vector species to increasing forest loss, which does not support the hypothesis that vector amplification as a consequence of increased host density occurs in response to deforestation, nor does it support the hypothesis that forest fragmentation leads to lower sandfly abundance by reducing the quality of vector habitat. However, for the most prevalent sandfly species (*Pa. aragaoi*), we found the same quadratic response to forest cover as with vertebrate hosts. While this species is not currently confirmed as a medically important species for the transmission of *Leishmania* to humans, multiple studies have confirmed the presence of *Leishmania* species in *Pa. aragaoi* samples and one recent study found a human blood meal from *Pa. aragaoi* [96]. Further evidence is needed to imply vector competence, such as evidence that the pathogen has breached the midgut barrier, so we classified this species as a possible vector. However, it is important to note that high abundance of this sandfly species has been correlated with proximity to armadillo holes and domestic animal dwellings [71].

The most consistent response of sandflies to deforestation was found in their response to increasing pasture cover. The probability of a sandfly pool containing any medically-important vector, or the dominant sandfly vector genus, *Nyssomyia* spp*.,* or the most prevalent species *Pa. aragaoi* was higher at sites surrounded by lower levels of cattle pasture (Fig 5). Potential mechanisms for this response include strong edge effects that reduce the quality of habitat. Further study is required to discern the likely causes of these edge effects, but possible scenarios include a decreased humidity at forest edges that can lead to habitat desiccation; diversionary feeding by sandflies on cattle such that fewer sandflies are host-seeking and available for capture; or even that sandflies can be negatively responding to the use of insecticide in cattle pastures.

The expansion of human activity into tropical forests can alter ecological communities and species interactions particularly at transition zones between forests and peri-urban areas [5,6], thereby potentially increasing the risk of infectious disease emergence from wildlife reservoirs and vectors into domestic vertebrate hosts and/or humans [4,7–9]. Our findings that both sandfly vectors and competent vertebrate hosts, including domestic dogs, are common where there is a matrix of both forest and croplands supports this mechanism of pathogen transmission. While our sampling scheme allowed us to sample across a landscape-scale deforestation gradient, it is important to note that we did not sample the extremes of vast tracts of continuous, undisturbed forests (compared with Kocher et al. [17]) nor entirely deforested landscapes lacking forest remnants. Instead, our study system represents a deforestation gradient resulting from rapid and recent forest conversion into primarily seed crop agriculture with much less hunting than typically occurs in systems fragmented by more dispersed small landholders. Consequently, while there was a gradient of deforestation across our study region, it is likely that the current forest landscape structure has not resulted in the same level of extirpation of vertebrate species that studies have documented in other southern Amazonian landscapes [144,145].

In summary, we found that vectors and their hosts had nuanced but consistent responses to deforestation; while overall sandfly abundance (including non-vectors) was unrelated to deforestation, sandfly vectors were more strongly associated with decreased amounts of cattle pasture, which was largely driven by the responses of the dominant sandfly taxa (*Pa. aragaoi* and *Nyssomyia* spp*.*). In contrast to sandflies, the probability of detecting any vertebrate, a vertebrate host, and domestic dogs in particular, was highest in bloodmeals from intermediate levels of forest cover. Additionally, samples that tested positive for the presence of *Leishmania* species also failed to show any response to our deforestation metrics, suggesting that *Leishmania* transmission can occur across both intact and degraded forests in this system. Individual samples that were positive for the presence of at least one *Leishmania* species showed the presence of both important domestic and sylvatic host species. In conclusion, we found the complex combined responses of vectors and hosts within the context of partly deforested landscapes did not support the naïve generality of the ‘dilution effect’ hypothesis. However, patterns of species responses to intermediate levels of deforestation likely play a key role in peridomestic transmission of *Leishmania* from the forest to human habitats and suggest consequences of forest conversion and increased human encroachment on disease transmission in this region of the Brazilian Amazon.

## Supporting information

S1 Table Site-level data for both the sandfly 12s metabarcoding data (vertebrate species) and the sandfly COI metabarcoding data (sandfly species). *N flies* and *N pools* denote the total number of individual flies collected at a site and the total number of pools they were divided into for a given site, respectively. *N pools post QC* is the number of pools included in the final dataset after quality control thresholds cleaned the metabarcoding data for both the sandfly 12s data and the sandfly COI data. A bracketed number below indicates the number of pooled samples processed with metabarcoding when this value was less than the total number of pooled samples available from a site. The *Total reads post QC* is the total number of assigned DNA sequence reads in the final dataset (also post quality control cleaning of metabarcoding data).
(DOCX)

S2 Table Site-level data for the number of sandfly pools that tested positive for the presence of *Leishmania* species.We used two primers in separate reactions to screen for *Leishmania* spp. (kDNA1 and Lbraz_kDNA3).
(DOCX)

S1 Fig Molecular workflow showing separate workflows for describing the sandfly and vertebrate communities from pooled sandfly samples.Our conservative quality control measures are outlined in the last boxes for each workflow to show how we reduced the risk of false positives of species presence and removed contaminating reads.
(DOCX)
