# Supplementary material for: Using iDNA to determine impacts of Amazonian deforestation on Leishmania hosts, vectors, and their interactions
Source: PLoS Negl Trop Dis. 2025 Mar 27;19(3):e0012925. doi: 10.1371/journal.pntd.0012925 (PMC11952761; doi:10.1371/journal.pntd.0012925)
Supplement: S1 Table — Site-level data for both the sandfly 12s metabarcoding data (vertebrate species) and the sandfly COI metabarcoding data (sandfly species). N flies and N pools denote the total number of individual flies collected at a site and the total number of pools they were divided into for a given site, respectively. N pools post QC is the number of pools included in the final dataset after quality control thresholds cleaned the metabarcoding data for both the sandfly 12s data and the sandfly COI data. A bracketed number below indicates the number of pooled samples processed with metabarcoding when this value was less than the total number of pooled samples available from a site. The Total reads post QC is the total number of assigned DNA sequence reads in the final dataset (also post quality control cleaning of metabarcoding data). (DOCX) [file pntd.0012925.s001.docx]

**S1 Table**

| ***Site*** | ***Sampling dates*** | ***N flies*** | ***N pools*** | ***N pools post QC 12s*** | ***Total reads post QC 12s*** | ***N pools post QC COI*** | ***Total reads post QC COI*** |
| --- | --- | --- | --- | --- | --- | --- | --- |
| A1 | 6.4 – 6.7.2015 | 496 | 10 | 0 | 0 | 10 | 200,215 |
| A2 | 6.4 – 6.7.2015 | 592 | 12 | 1 | 580 | 10 | 99,583 |
| A3 | 6.4 – 6.7.2015 | 3061 | 61 | 23 | 48,337 | 55 | 855,619 |
| A4 | 6.4 – 6.7.2015 | 4602 | 92 | 17 | 29,518 | 83 | 867,839 |
| B5 | 6.15 – 6.18.2015 | 8469 | 170 | 45  [90] | 583,567 | 69  [70] | 2,155,303 |
| B6 | 6.15 – 6.18.2015 | 997 | 20 | 8 | 82,525 | 20 | 926,329 |
| B7 | 6.15 – 6.18.2015 | 981 | 20 | 7 | 172,855 | 20 | 244,933 |
| B8 | 6.15 – 6.18.2015 | 688 | 14 | 8 | 59,767 | 6 | 30,294 |
| C9 | 6.22 – 6.25.2015 | 789 | 16 | 10 | 110,510 | 15 | 83,126 |
| C10 | 6.22 – 6.25.2015 | 584 | 12 | 9 | 21,211 | 12 | 428,591 |
| C11 | 6.22 – 6.25.2015 | 273 | 6 | 5 | 76,527 | 6 | 606,585 |
| C12 | 6.22 – 6.25.2015 | 468 | 9 | 4 | 43,667 | 9 | 933,400 |
| D13 | 7.24 – 7.27.2015 | 810 | 16 | 10 | 120,428 | 15 | 683,643 |
| D14 | 7.24 – 7.27.2015 | 645 | 13 | 5 | 13,197 | 13 | 282,486 |
| D15 | 7.24 – 7.27.2015 | 659 | 13 | 7 | 73,613 | 13 | 538,961 |
| E16 | 7.24 – 7.27.2015 | 342 | 7 | 6 | 97,697 | 6 | 55,631 |
| E17 | 7.17 – 7.20.2015 | 400 | 8 | 7 | 51,021 | 7 | 197,784 |
| E18 | 7.17 – 7.20.2015 | 248 | 5 | 3 | 17,667 | 5 | 87,331 |
| E19 | 7.17 – 7.20.2015 | 131 | 3 | 2 | 31,204 | 3 | 35,766 |
| F21 | 5.04 – 5.07.2016 | 2691 | 54 | 26 | 88,285 | 48  [50] | 1,292,681 |
| F22 | 5.04 – 5.07.2016 | 7780 | 155 | 41  [90] | 445,580 | 54  [70] | 370,921 |
| F23 | 5.04 – 5.07.2016 | 909 | 18 | 16 | 110,649 | 17 | 380,492 |
| F24 | 5.04 – 5.07.2016 | 234 | 5 | 3 | 6,108 | 5 | 68,530 |
| G25 | 4.27 – 4.30.2016 | 2534 | 50 | 24  [34] | 392,974 | 45 | 1,568,407 |
| G26 | 4.27 – 4.30.2016 | 503 | 10 | 9 | 175,540 | 10 | 433,076 |
| G27 | 4.27 – 4.30.2016 | 3136 | 63 | 36 | 338,316 | 50 | 1,035,287 |
| G28 | 4.27 – 4.30.2016 | 1457 | 29 | 18 | 127,805 | 28 | 727,791 |
| H29 | 5.11 – 5.14.2016 | 1778 | 36 | 26 | 447,049 | 33 | 804,341 |
| H30 | 5.11 – 5.14.2016 | 685 | 14 | 9 | 87,953 | 13 | 341,998 |
| H31 | 5.11 – 5.14.2016 | 569 | 11 | 8 | 159,673 | 10 | 176,840 |
| H32 | 5.11 – 5.14.2016 | 2153 | 43 | 18 | 237,533 | 41 | 338,660 |
| I33 | 6.8 – 6.11.2016 | 481 | 10 | 7 | 96,251 | 10 | 361,086 |
| I34 | 6.8 – 6.11.2016 | 946 | 19 | 6 | 45,351 | 18 | 163,266 |
| I35 | 6.8 – 6.11.2016 | 186 | 4 | 1 | 1,994 | 4 | 16,861 |
| I36 | 6.8 – 6.11.2016 | 303 | 6 | 3 | 48,434 | 6 | 74,353 |
| J37 | 5.23 – 5.26.2016 | 3342 | 67 | 38 | 279,387 | 51  [56] | 850,245 |
| J38 | 5.23 – 5.26.2016 | 268 | 5 | 2 | 1,692 | 4 | 55,900 |
| J39 | 5.23 – 5.26.2016 | 768 | 15 | 6 | 19,638 | 11 | 416,710 |
| J40 | 5.23 – 5.26.2016 | 816 | 16 | 7 | 9,670 | 16 | 544,092 |
