# Supplementary material for: Using iDNA to determine impacts of Amazonian deforestation on Leishmania hosts, vectors, and their interactions
Source: PLoS Negl Trop Dis. 2025 Mar 27;19(3):e0012925. doi: 10.1371/journal.pntd.0012925 (PMC11952761; doi:10.1371/journal.pntd.0012925)
Supplement: S2 Table — Site-level data for the number of sandfly pools that tested positive for the presence of Leishmania species.We used two primers in separate reactions to screen for Leishmania spp. (kDNA1 and Lbraz_kDNA3). (DOCX) [file pntd.0012925.s002.docx]

**S2 Table**

| *Site* | *N pools screened* | *N pools positive for Leishmania using kDNA1* | *N pools positive for Leishmania using Lbraz_kDNA3* |
| --- | --- | --- | --- |
| A1 | 10 | 0 | 0 |
| A2 | 12 | 0 | 0 |
| A3 | 61 | 0 | 0 |
| A4 | 92 | 1 | 0 |
| B5 | 170 | 4 | 3 |
| B6 | 20 | 0 | 0 |
| B7 | 20 | 3 | 0 |
| B8 | 14 | 0 | 0 |
| C9 | 16 | 0 | 0 |
| C10 | 12 | 1 | 0 |
| C11 | 6 | 0 | 0 |
| C12 | 9 | 0 | 0 |
| D13 | 16 | 0 | 0 |
| D14 | 13 | 0 | 0 |
| D15 | 13 | 0 | 0 |
| E16 | 7 | 1 | 0 |
| E17 | 8 | 0 | 0 |
| E18 | 5 | 0 | 0 |
| E19 | 3 | 2 | 1 |
| F21 | 54 | 4 | 0 |
| F22 | 155 | 1 | 1 |
| F23 | 18 | 0 | 0 |
| F24 | 5 | 0 | 0 |
| G25 | 50 | 0 | 0 |
| G26 | 10 | 0 | 0 |
| G27 | 63 | 3 | 1 |
| G28 | 29 | 0 | 1 |
| H29 | 36 | 5 | 2 |
| H30 | 14 | 0 | 0 |
| H31 | 11 | 0 | 1 |
| H32 | 43 | 0 | 2 |
| I33 | 10 | 1 | 1 |
| I34 | 19 | 2 | 1 |
| I35 | 4 | 0 | 0 |
| I36 | 6 | 0 | 1 |
| J37 | 67 | 0 | 1 |
| J38 | 5 | 0 | 0 |
| J39 | 15 | 0 | 0 |
| J40 | 16 | 1 | 0 |
